# Adult bone marrow mesenchymal and neural crest stem cells are chemoattractive and accelerate motor recovery in a mouse model of spinal cord injury

**DOI:** 10.1186/s13287-015-0202-2

**Published:** 2015-11-04

**Authors:** Virginie Neirinckx, Gulistan Agirman, Cécile Coste, Alice Marquet, Valérie Dion, Bernard Rogister, Rachelle Franzen, Sabine Wislet

**Affiliations:** Groupe Interdisciplinaire de Génoprotéomique Appliquée (GIGA), Neurosciences Research Center, Unit of Nervous system disorders and treatment, University of Liège, Tour de Pathologie 2, Avenue de l’Hôpital, 1, 4000 Liège, Belgium; GIGA, Development, Stem Cells and Regenerative Medicine Research Center, University of Liège, Liège, Belgium; Neurology Department, University Hospital, Liège, Belgium

## Abstract

**Introduction:**

Stem cells from adult tissues were considered for a long time as promising tools for regenerative therapy of neurological diseases, including spinal cord injuries (SCI). Indeed, mesenchymal (MSCs) and neural crest stem cells (NCSCs) together constitute the bone marrow stromal stem cells (BMSCs) that were used as therapeutic options in various models of experimental SCI. However, as clinical approaches remained disappointing, we thought that reducing BMSC heterogeneity should be a potential way to improve treatment efficiency and reproducibility.

**Methods:**

We investigated the impact of pure populations of MSCs and NCSCs isolated from adult bone marrow in a mouse model of spinal cord injury. We then analyzed the secretome of both MSCs and NCSCs, and its effect on macrophage migration *in vitro*.

**Results:**

We first observed that both cell types induced motor recovery in mice, and modified the inflammatory reaction in the lesion site. We also demonstrated that NCSCs but especially MSCs were able to secrete chemokines and attract macrophages *in vitro*. Finally, it appears that MSC injection in the spinal cord enhance early inflammatory events in the blood and spinal cord of SCI mice.

**Conclusions:**

Altogether, our results suggest that both cell types have beneficial effects in experimental SCI, and that further investigation should be dedicated to the regulation of the inflammatory reaction following SCI, in the context of stem cell-based therapy but also in the early-phase clinical management of SCI patients.

**Electronic supplementary material:**

The online version of this article (doi:10.1186/s13287-015-0202-2) contains supplementary material, which is available to authorized users.

## Introduction

Regenerative processes are relatively modest in the mammalian central nervous system (CNS), and stem cell-based therapy has raised important hopes for treating neurological diseases for many years. Indeed, patients suffering from degenerative or traumatic pathologies of the nervous system have to deal with heavy clinical, social and psychological impairments, while only compensatory attempts could be considered to reduce symptoms. Stem cells from adult bone marrow stroma have been proposed as highly powerful candidates in such therapies and considered worldwide in a large panel of animal models for various neurological conditions, such as Parkinson’s disease, Alzheimer’s disease, Huntington’s disease, multiple sclerosis, amyotrophic lateral sclerosis, stroke, or traumatic spinal cord injuries (reviewed in [1]). Beyond their debated ability to transdifferentiate into neural cells, bone marrow stromal stem cells (BMSCs) effectively show interesting features, such as immunomodulating [2], neurotrophic [3], or pro-angiogenic activities [4]. These stem cells were shown to efficiently enhance functional recovery in experimental models of spinal cord injuries (SCI) by modulating diverse physiopathological events, such as inflammatory reaction, oxidative stress, apoptosis, angiogenesis, axonal growth, or myelination (reviewed in [5]). BMSCs have subsequently been considered in a clinical context for treating SCI patients [6]. If only modest enhancement was observed, additional investigation is required to identify the cellular and molecular mechanisms underlying BMSC activity in the injured spinal cord, in order to further improve patient outcome. Noteworthy, the lack of exact phenotypic characterization of BMSCs (due to the absence of specific membrane markers and non-standardized culture methods) prompts supplemental difficulty [7, 8]. Furthermore, it has been demonstrated that the classically defined mesenchymal stem cells (MSCs) from adult bone marrow are actually arising from different embryonic lineages. Indeed, although adult BMSCs were commonly considered to be of mesodermal origin [9], several studies have conclusively shown that a subset of adult BMSCs derives from the neural crest [10, 11]. Our group previously reported a complete comparison of bone marrow MSCs and neural crest stem cells (NCSCs), and showed that both cell types were able to give rise to functional neurons *in vitro* [12]. Even if those cells failed to differentiate into neurons in experimental models for Parkinson’s disease [13–16] and other neurological conditions such as SCI [1, 17, 18], BMSCs are still considered as powerful candidates for cell therapy protocols. Indeed, the literature largely associates the positive impact of BMSCs in neurological disorders to their secretome, composed of all the molecules and vesicles secreted by these cells. BMSC secretome is enriched in growth and neurotrophic factors, cytokines/chemokines, angiogenic factors, etc., and could be extremely interesting from a therapeutic perspective. Many studies have identified secretome-related effects of BMSCs *in vitro,* but also in animal models for different CNS pathologies, including SCI [19–22]. Among other properties, BMSCs are able to sense and modulate inflammatory reaction [23, 24] and are, for instance, already used to reduce immune rejection in graft-versus-host disease [25].

The aim of this study was to compare adult bone marrow MSCs and NCSCs properties, both *in vitro* and *in vivo*, in the context of SCI-related inflammation. Indeed, traumatic SCI encompasses many physiopathological events including an intense inflammatory reaction that starts directly after the lesion and persists chronically for weeks to months [26]. Consequently, for many years, SCI patients received methylprednisolone in attempt to limit lesion extent and swelling as early as possible [27]. However, it is now well accepted that beyond the classical view about detrimental post-traumatic neuroinflammation, the immune system displays multiple neuroprotective functions and requires a subtle regulation [28, 29]. Systemic immune cells, such as granulocytes [30] or monocytes/macrophages [31], play important roles in CNS tissue regeneration after trauma. Macrophages are the most studied immune cells in the context of SCI and have already been tested in SCI patients [32]. In addition, experimental data recently demonstrated that different categories of macrophages (with distinct phenotypes or origins) have distinct roles in SCI recovery [33–35].

The main objective of this paper was, therefore, to study the immunomodulatory properties of both MSCs and NCSCs, and their respective secretomes. We aimed to highlight how each cell type could manage functional recovery after SCI, by modulating the recruitment of immune cells from the systemic blood towards the injured spinal cord.

## Methods

### Animal handling

Eight- to ten-week-old Wnt1*-*Cre*/*R26R-*LacZ* double transgenic mice [36] (obtained by mating C57Bl/6 J Wnt1-Cre mice [37] and C57BL ROSA26R-LacZ mice [38]) were used to isolate NCSC and MSC clones from adult bone marrow stromal cell cultures (BMSCs). Ten- to twelve-week-old wild type C57BL/6 J female mice were used as recipient mice for graft experiments after spinal cord injury, in order to facilitate bladder emptying and avoid urinary tract infections. Menstrual cycle was controlled at the day of surgery. Animals were bred at the University of Liège Central Animal facility. This study was approved by the Ethics Committee of the Medicine Faculty of the University of Liège (ethical permit 1200), and experiments were performed in accordance with the rules set by this committee and the Swiss Academy of Medical Sciences.

### Cell culture, clonal selection of MSCs and NCSCs, and preparation of MSC- and NCSC-conditioned medium

Eight- to ten-week-old Wnt1*-*Cre*/*R26R-*LacZ* double transgenic mice [36] were used to isolate NCSC and MSC clones from adult bone marrow stromal cell cultures (BMSCs), obtained from femoral and tibial aspirations and resuspended in MesenCult Medium (MesenCult, Stem Cells Technologies, Grenoble, France). After 24 h, non-adherent cells were removed. After reaching confluence, BMSCs were dissociated with 0.05 % trypsin-EDTA (Life Technologies, Carlsbad, CA, USA) and then sub-cultured (750,000 cells/25 cm^2^) at 37 °C, in a 95 % O_2_/5 % CO_2_ atmosphere. For clonal selection, passage 5-BMSCs were seeded in a 96-well plate at a mean dilution of 0.7 cell/well, in MesenCult Medium. Based on β-galactosidase expression, we selected five clonal populations of NCSCs and five clonal populations of MSCs. At confluence, cells were dissociated with 0.05 % trypsin-EDTA and sub-cultured under the same conditions [12]. For conditioned medium (CM) preparation, two cultures of 500,000 cells were prepared, respectively containing five NCSC clones (NCSC_mix_) and four MSC clones (MSC_mix_) in equal number. Cell mixes were placed overnight at a density of 2,000 cells/cm^2^ in 5 mL of MesenCult (alone or supplemented with 1 μg/mL of *Escherichia coli* lipopolysaccharide (E. coli LPS 055:B5, L2880, Sigma-Aldrich, Saint-Louis, MO, USA)). Cells were then rinsed three times with 5 mL PBS, and MesenCult medium was replaced by 5 mL serum-free DMEM for 24 h. After centrifugation and 0.22 um filtration, MSC-CM, ^LPS^MSC-CM, NCSC-CM and ^LPS^NCSC-CM were stored at -20 °C.

### Cytokine array and ELISA experiments

For the qualitative and quantitative analysis of MSC-CM and NCSC-CM, Mouse Cytokine Array (ARY006, R&D Systems, Minneapolis, MN, USA) and Mouse Quantikine –G-CSF, M-CSF, CXCL1, CXCL2, CXCL10, CXCL12, IL-6, CCL2, CCL5, sICAM-1, and TIMP-1- ELISA kits (R&D Systems, Minneapolis, MN, USA) were respectively performed with conditioned medium samples. Spinal cord and plasma samples were also processed using these assays, according to the manufacturer’s suggested procedure.

### Chemotaxis and metabolic assays – migration of RAW264.7 macrophages in response to MSC- or NCSC-conditioned medium

RAW264.7 macrophage cell line was used to test for the chemoattractant power of MSC-CM or NCSC-CM. The RAW264.7 cells were cultured in DMEM containing 10 % decomplemented fetal bovine serum. After being labeled with Cell Tracker Green (CTG) (Life Technologies, Carlsbad, CA, USA) in serum-free DMEM, 100,000 RAW264.7 cells were placed on 5.2 mm-diameter filters (each containing 100,000 5 um-pores) (ChemoTx, NeuroProbe, Gaithersburg, MD, USA), above a bottom chamber containing 30 μL of MSC-CM, ^LPS^MSC-CM, NCSC-CM and ^LPS^NCSC-CM. The plate was incubated at 37 °C for 20 h. After incubation, non-migrating macrophages were removed from the top of the filter, and we quantified the percentage of filter area occupied by macrophages that migrated throughout the filter, in response to MSC-, ^LPS^MSC-, NCSC- or ^LPS^NCSC-CM. Metabolic assay was performed using tetrazolium compound-based CellTiter 96H AQueous One Solution Cell Proliferation (MTS) assay (Promega, Madison, WI, USA). A total of 10,000 RAW264.7 cells were seeded into wells of a 96-well plate. After 20 h of culture in serum-free DMEM or in the presence of 1 μg/mL LPS, MSC-CM, and NCSC-CM (with or without LPS pretreatment), a MTS assay was performed according to the manufacturer’s instructions. Basically, after conversion of MTS into colored formazan, absorbance of each well was evaluated at 490 nm. Each experiment was performed in triplicate and repeated three times (*n* = 3).

### Spinal cord injury and cell transplantation experiment

Wild type C57BL/6 J female mice (10- to 12-weeks old) were used as recipient mice for graft experiments after spinal cord injury. Just before the surgery, two cell suspensions containing, respectively, five NCSC clones and four MSC clones in equal numbers were prepared. Whereas NCSC_mix_ were already traceable thanks to their β-galactosidase activity, we needed to label MSC_mix_ with CTG to allow their traceability *in vivo*. The spinal cord injury procedure and cell graft were performed as previously described [39]. Briefly, mice were anesthetized with ketamine/xylazine and kept on a warm pad to undergo T12 laminectomy. After being subjected to a moderate (50 kDyn) T11/T12 contusion, SCI (IH-0400 Impactor, Precisions Systems and Instrumentation, Fairfax, VA, USA), mice immediately received three intralesional injections of 1 × 10^4^ cells, inside the contusion site, 1 mm above, and 1 mm below, respectively. This early timing of transplantation was purposely selected in order to allow grafted cell-related early neuroprotective and immunomodulatory effects to occur. After the surgery, mice received saline injections to compensate for blood loss and were placed in temperature-controlled incubators until their complete awakening. Bladders were manually emptied two times daily until spontaneous voiding returned.

### Harvesting of spinal cord tissue and blood

Fresh spinal cord extract and whole blood were obtained 24 h and 7 days post-injury (dpi). Animals were euthanized with a lethal dose of pentobarbital. Blood samples were obtained by cardiac puncture and plasma was collected into EDTA tubes. Spinal cord segments were immediately removed (0.5 cm centered on the lesion site) and placed into ice-cold PBS, before protein extraction and western blot analyses. For histological analyses of spinal cords, mice were sacrificed at 28 dpi by intracardiac perfusion of ice-cold PBS, followed by paraformaldehyde (PFA) 4 % (in PBS 0.1 M). Spines were dissected and cords were immediately removed, post-fixed for 2 h at 4 °C in the same fixative then immersed overnight in a solution of sucrose 20 % (in PBS 0.1 M). Longitudinal 20 μm-sections were cut and stored at −20 °C.

### Cell and tissue staining

#### Immunostainings

Briefly, slices (or cells) were incubated for 1 h with 10 % normal donkey serum in PBS 0.1 M (supplemented with 0.3 % Triton X-100 for intracellular antigens). For specific immunofluorescent staining, anti-nestin (1:300, NB100-1604, Novus Biologicals, Littleton, CO, USA), anti-Sox2 (1:250; sc-17320, Santa Cruz, Dallas, Texas, USA), anti p75NTR (1:200, AB1554, Millipore, Darmstadt, Germany), anti-Sca-1 (1:100, ab25195, Abcam, Cambridge, United Kingdom), anti-arginase 1 (1:100, 610708 BD Biosciences, Franklin Lakes, NJ, USA), anti-Iba1 (1:800, 019-19741, Wako Chemicals, Richmond, VA, USA), anti-GFAP (1:1000, 20334, Dako, Glostrup, Denmark), anti-laminin (1:200, L9393, Sigma-Aldrich, Saint-Louis, MO, USA) were diluted in PBS 0.1 M overnight at 4 °C. After PBS washes, brain sections were incubated at room temperature with fluorescein and rhodamine red X-conjugated secondary antibodies (1:500; Jackson Immunoresearch Laboratories, Westgrove, PA, USA) or with peroxidase-coupled secondary antibodies (1:500, Dako, Glostrup, Denmark) and diaminobenzidine revelation. Nuclei were counterstained with Hoescht for fluorescent staining. Image acquisition and analysis were performed using a Zeiss AxioImager Z1 epifluorescent microscope coupled with FluoView (Olympus), and Olympus AX-70 microscope coupled with AnalySIS software (Olympus).

#### Luxol Fast Blue-Neutral Red

Spinal cord sections were incubated in 0.0125 % Luxol Fast Blue, as previously described [40]. Lesion volume was evaluated by collecting maps of 15 sections and performing 3D reconstructions via Mercator/Map3D software (ExploraNova).

#### X-gal/hematoxylin staining

Stainings were performed as we previously described [13].

### Western-blot analysis

Proteins were extracted from spinal cord segments collected at 24 h and 7 days post-injury, resolved in Novex 4-12 % BisTris gels (NuPage, Life Technologies, Carlsbad, CA, USA), and transferred onto a PVDF membrane (Roche, Basel, Switzerland) according to standard protocols. Blots were then probed with primary antibodies targeting Ly6G/Gr-1 (1/500, MCA2387, AbD Serotec, Kidlington, United Kingdom) and CD11b (1/500, ab75476, Abcam, Cambridge, United Kingdom) at 4 °C overnight. Secondary antibodies coupled to HRP (1/3000, ab97057, Abcam, Cambridge, United Kingdom) were applied for 1 h, and blots were revealed with a chemiluminescent substrate (ThermoScientific, Waltham, MA, USA) and imaged with the ImageQuant 350 scanning system (cooled-CCD camera, GE Healthcare, Little Chalfont, Buckinghamshire, United Kingdom). α-tubulin detection (1/10.000, ab56676, Abcam, Cambridge, United Kingdom) was used as internal standard. Expression levels were quantified with ImageMaster 1D Prime Software (GE Healthcare, Little Chalfont, Buckinghamshire, United Kingdom).

### Statistical analysis

Data were analyzed statistically using the Statistica 10 program (StatSoft, Tulsa, OK, USA). Results are reported as mean ± standard error of the mean, with the n described as the number of mice in each group. Level of statistical significance was set at *p* < 0.05.

## Results

### *In vitro* characterization of MSCs and NCSCs from Wnt1-CRE/R26R-*LacZ* mice bone marrow

Bone marrow stromal cells (BMSCs) have already been considered in a wide variety of animal models for neurological diseases including spinal cord injury (SCI). Beneficial effects are globally reported, despite a well-known experimental variability. One explanation concerning this variability would be the heterogeneity of bone marrow cell populations. Indeed, BMSCs are composed of multiple progenitors and stem cell types with different embryonic origins. In order to address this variability problem and to better define the specific role of bone marrow mesenchymal stem cells (MSCs) and bone marrow neural crest stem cells (NCSCs) in tissue regeneration after SCI, we decided to investigate pure populations of MSCs and NCSCs separately. As previously described, we isolated both cell types from bone marrow stromal cells from adult Wnt1-CRE/R26R-*LacZ* mice [36], which allowed us to specifically discriminate neural crest cells (which express *LacZ*) among the whole BMSC population.

**Fig. 1:**
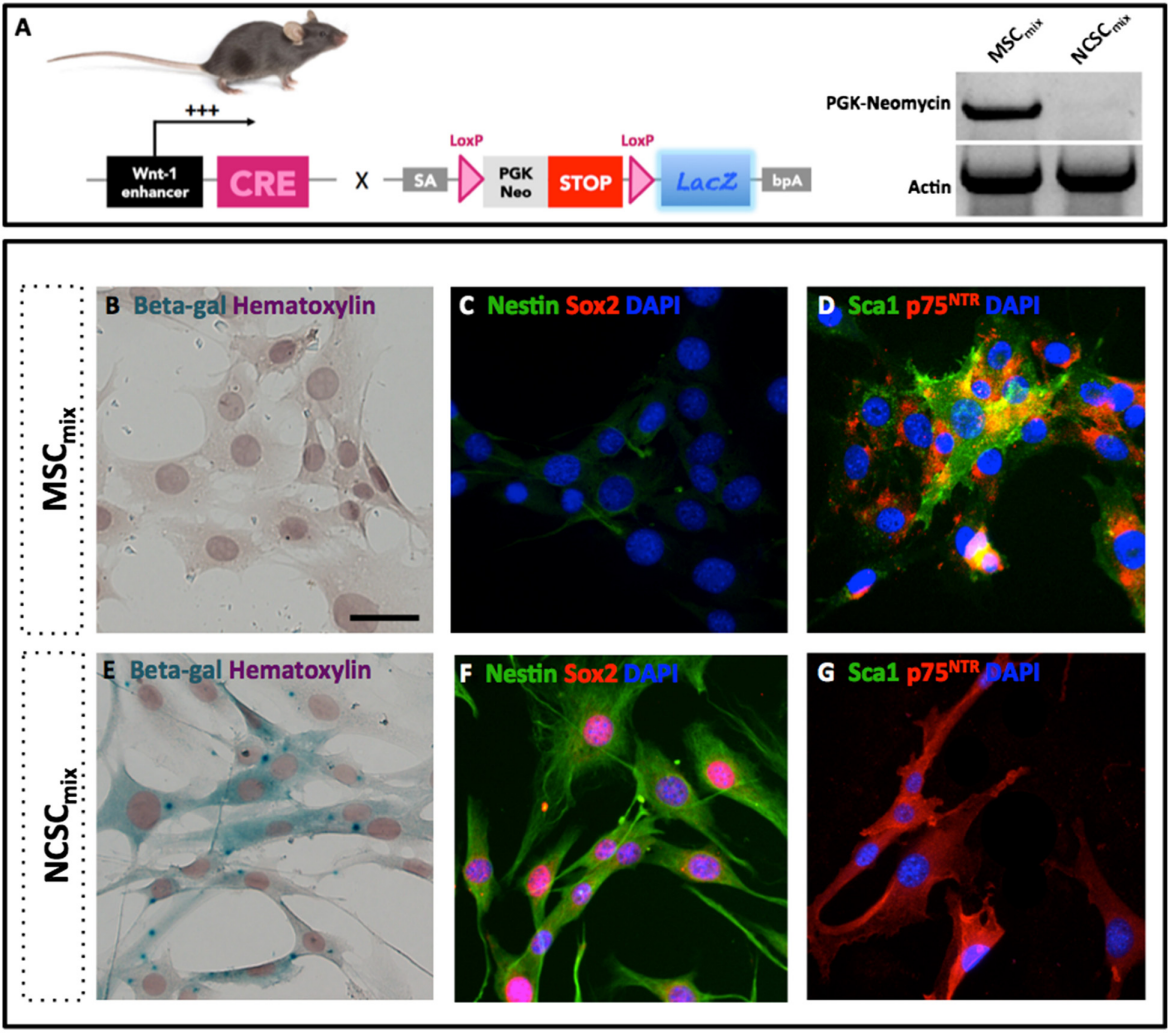
Characterization of MSC_mix_ and NCSC_mix_ isolated from the bone marrow of adult Wnt1-CRE/R26R-LacZ mice. After recombination, NCSCs from Wnt1-CRE/R26R-LacZ mice express LacZ gene. MSCs did not undergo Cre/Lox recombination and conserved the PGK-Neo cassette (**a**). MSC_mix_ are adherent fibroblast-like cells, do not express β-galactosidase (**b**) or Sox2 **c** (red), slightly express Nestin (**c**) (green), p75NTR (**d**) (red), and Sca-1 (**d**) (green). NCSC_mix_ have a similar morphology, express β-galactosidase (**e**), Nestin (**f**) (green), Sox2 (**f**) (red) and p75NTR (**g**) red), but not Sca-1(**g**) (green). Scale bar = 20 μm. *MSC* mesenchymal stem cell, *NCSC* neural crest stem cell

Since no specific marker would allow us to perform a purification protocol to isolate MSCs and NCSCs from adult bone marrow, we used a clonal selection method. In order to limit the risk of clonal effect and rather get a global population effect, four MSCs and five NCSCs clones were, therefore, cultivated separately and respectively pooled together (in equal number) as MSC_mix_ and NCSC_mix_, before each experiment. We first confirmed that NCSCs underwent CRE-lox recombination and lost PGK-Neomycin cassette, and that MSCs conserved it (Fig. 1a). Despite a similar morphology, NCSCs and MSCs exhibit different phenotypes. NCSC_mix_ (β-galactosidase-positive, Fig. 1e) expressed high levels of Nestin, Sox2 (Fig. 1f) and P75^NTR^ (Fig. 1g), whereas MSC_mix_ were β-galactosidase-negative (Fig. 1b) and Sox2-negative (Fig. 1c), and only display low expression of Nestin (Fig. 1c), and P75^NTR^ markers (Fig. 1d), while expressing Sca-1/Ly6 membrane receptor (Fig. 1d).

### MSCs and NCSCs accelerate locomotor recovery in mice with moderate spinal cord contusion injury

**Fig. 2:**
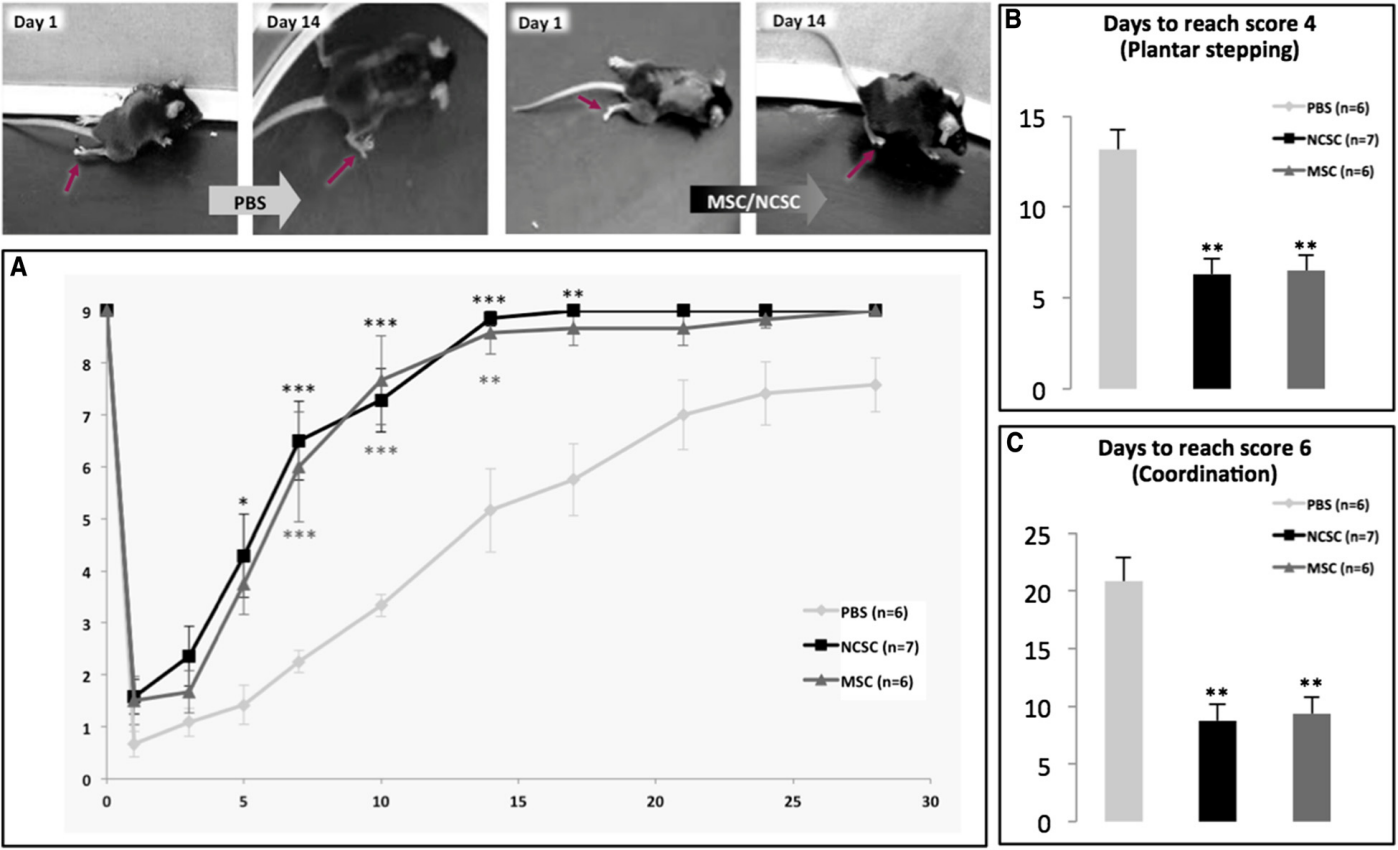
MSC_mix_ and NCSC_mix_ intraspinal cell graft accelerates motor recovery in mice with moderate SCI. MSC-grafted mice and NCSC-grafted mice recovered hindlimb motility faster than PBS-injected mice, as reflected by the Basso Mouse Scale scoring (**a**) (*n* = 6 to 7, repeated measures ANOVA and HSD post-test, *p < 0.05; **p < 0.01; ***p < 0.001). Indeed, it appears that MSC- and NCSC-grafted mice reach score 4 (plantar stepping, **b**) and score 6 (coordination, **c**) in a significantly reduced time interval (*n* = 6 to 7, one-way ANOVA and HSD post-test, **p < 0.01). *MSC* mesenchymal stem cell, *NCSC* neural crest stem cell, *SCI* spinal cord injury, *PBS* phosphate-buffered saline, *ANOVA* analysis of variance, *HSD* honestly significant difference

Many experimental studies have so far documented the high potential of BMSCs in the treatment of experimental SCI, but unsatisfactory clinical applications prompted us to further investigate the different mechanisms induced by those cells. After characterizing two different subsets of stem cells among the heterogeneous BMSC population, we wanted to define the role MSCs and NCSCs could specifically play in the treatment of SCI and the management of SCI-induced inflammatory reaction. We, therefore, decided to inject MSC_mix_ or NCSC_mix_ in a mouse model for contusive SCI. MSC_mix_ or NCSC_mix_ were intraspinally transplanted directly after a thoracic contusion injury in C57BL/6 J adult mice, and PBS was injected as vehicle. Under those conditions, we observed that MSC_mix-_ and NCSC_mix_-transplanted mice significantly recovered hindlimb motility starting from day 5 post-injury compared to control mice (Fig. 2a) (*n* = 6 to 7, repeated measures ANOVA and HSD post-test, *p < 0.05; **p < 0.01; ***p < 0.001), according to the Basso Mouse Scale [41]. We observed that the time to reach score 4 (corresponding to plantar stepping) was reduced in MSC_mix_- (6.50 ± 0.80 days, **p < 0.01) and NCSC_mix_-injected mice (6.28 ± 0.83 days, **p < 0.01) compared to PBS-injected control mice (13.16 ± 1.10 days) (Fig. 2b). Similarly, the time to reach score 6 (corresponding to motor coordination) was significantly shortened in MSC_mix_- (9.33 ± 1.47 days, **p < 0.01) and NCSC_mix_-injected mice (8.71 ± 1.39 days, **p < 0.01) compared to controls (20.83 ± 2.02 days) (Fig. 2c) (*n* = 6 to 7, one-way ANOVA and HSD post-test). Finally, 28 days post-injury, control mice reached a BMS score that came closer to the score of the cell-injected groups. However, these results highlight a significantly accelerated motor recovery of mice that received MSC_mix_ or NCSC_mix_ transplantation, which should be considered of significant interest from a clinical point of view.

### MSCs and NCSCs transplantation modify the lesion environment 28 days post-SCI

**Fig. 3:**
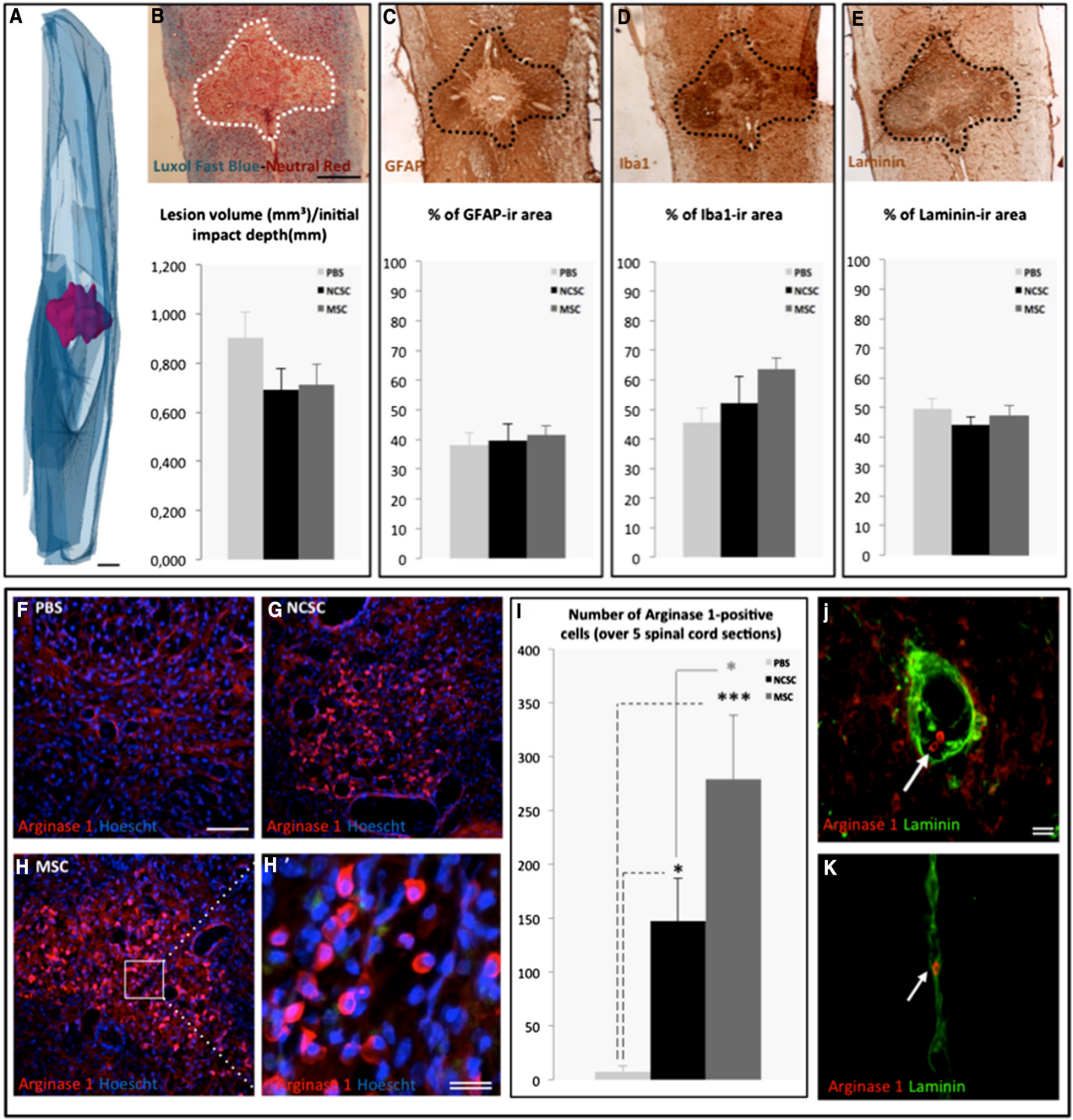
MSC_mix_ and NCSC_mix_ intraspinal cell graft influences lesion environment. After 3D reconstruction of spinal cord sections (**a**) stained with Luxol Fast Blue-Neutral Red (**b**), we evaluated the lesion volume in each group, which tends to be reduced in NCSC- (black) and MSC-grafted mice (dark grey) compared to controls (light grey) (**b**). We translated the evaluated lesion area on the next adjacent section to quantify the expression of GFAP, Iba1 and laminin. The GFAP-immunoreactive (ir) (**c**) and laminin-ir area (**e**) did not differ between the three groups, but the Iba1-ir area was a bit increased in MSC-grafted spinal cord sections (**d**). We also observed the presence of arginase 1-ir cells, not in control mice (**f**) but only in NCSC- (**g**) and MSC-grafted mice (**h**). The little rounded cells (h’) were more abundant in MSC- than in NCSC-grafted mice (**i**), and were recovered close to laminin-ir blood vessels (**j**), (**k**). (*n* = 6 to 7, one-way ANOVA and HSD post-test, *p < 0.05; ***p < 0.01). Scale bar = 500 μm (a to e), 200 μm (f to h), 20 μm (double, h’, j, k). *MSC* mesenchymal stem cell, *NCSC* neural crest stem cell, *GFAP* glial fibrillary acidic protein, *ANOVA* analysis of variance, *HSD* honestly significant difference

Four weeks after the injury/transplantation procedure, we were not able to retrieve any β-galactosidase-positive NCSCs or CTG-labeled MSCs. Indeed, it is well known that BMSC integration and survival into the host spinal cord tissue of experimental rodent models are very low [42]. Still, we evaluated the size of SCI lesions in order to attest to a protective effect of MSC and NCSC transplantation that could be associated with an improved locomotor recovery. We stained 28-day longitudinal spinal cord sections with Luxol Fast Blue/Neutral Red [40] and selected the lesioned area (Fig. 3b). Sections were then assembled to obtain a 3D volume of the lesion (Fig. 3a), which was then normalized to the initial impact displacement, provided by the impactor software. We observed that the lesion size tend to decrease in MSC_mix_- and NCSC_mix_-grafted mice (in accordance with the ameliorated locomotion) (Fig. 3b). We applied the same selection of lesioned area on the directly adjacent sections, and noticed that the percentage of GFAP-immunoreactive (ir) (astroglial scar) and laminin-ir area (blood vessels) in the lesion zone was similar between the three groups (Fig. 3c, e) (*n* = 6 to 7, one-way ANOVA, *p* > 0.05). Conversely, the percentage of Iba1-ir area (macrophages) tended to increase in sections corresponding to MSC_mix_-grafted spinal cords (Fig. 3d) (*n* = 6 to 7, one-way ANOVA and HSD post-test, *p* = 0.1).

Based on these results, we analyzed the expression of arginase-1 which characterizes the anti-inflammatory phenotype of myeloid immune cells [43]. We also had to take into consideration that arginase-1 was also expressed by different cell types in the spinal cord, [44], but fortunately, as observed in our control condition (Fig. 3f), the basal expression level of arginase-1 was close to background. In this context, we detected arginase-1-ir cells in the epicenter of spinal cord lesions from MSC_mix_- (Fig. 3h) and NCSC_mix_-grafted mice (Fig. 3g), in a significantly higher number compared to control mice (Fig. 3i) (*n* = 6 to 7, one-way ANOVA and HSD post-test, *p < 0.05; ***p < 0.01). We quantified little rounded cells that expressed a high level of arginase-1. These rounded arginase-1-ir cells (Fig. 3h’) were also found close to laminin-ir blood vessels (Fig. 3j, k).

At this point of the study, we observed that even if grafted cells were not recovered in the spinal cord tissue, they apparently worked on the inflammatory environment of the lesion and promoted an accelerated motor recovery of SCI mice.

### Characterization of MSC- and NCSC-secreted chemokines and cytokines under normal and inflammatory conditions

**Fig. 4:**
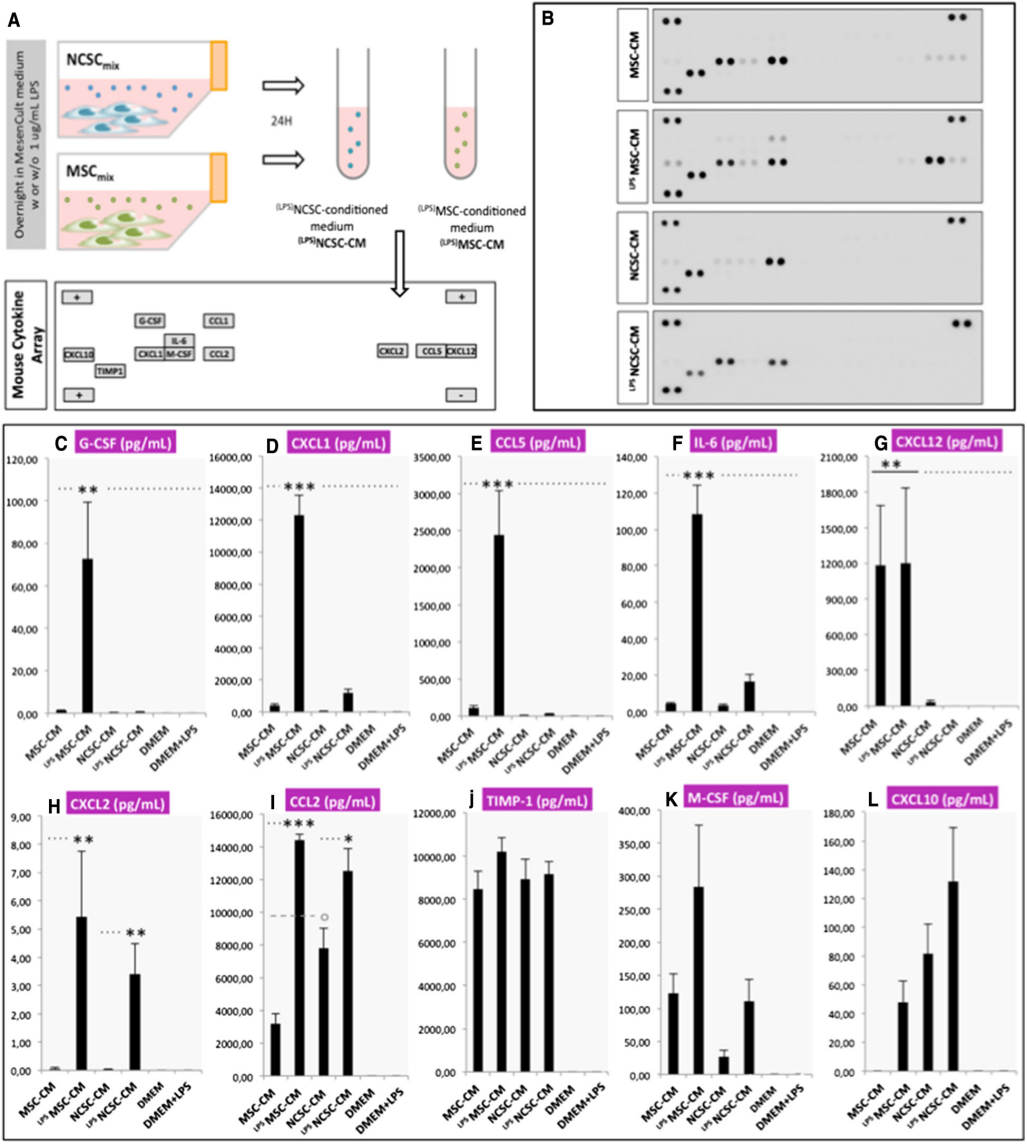
Qualitative and quantitative analysis of MSC_mix_- and NCSC_mix_-secretome. NCSC_mix_- and MSC_mix_-conditioned media (CM) are prepared by culturing MSC_mix_ and NCSC_mix_ in serum-free DMEM for 24 h (with or without stimulation by 1 μg/mL LPS: ^LPS^MSC-CM and ^LPS^NCSC-CM) and analyzed by *Mouse Cytokine Array* (**a**). Qualitative analysis of arrays revealed the presence of different chemokines and cytokines, especially present in MSC-secretome (**b**), and their concentration in each CM sample was assessed by ELISA assays. We observed an increased concentration of G-CSF (**c**), CXCL1 (**d**), CCL5 (**e**), IL-6 (**f**) in ^LPS^MSC-CM, whereas CXCL12 was present in both MSC-CM and ^LPS^MSC-CM (**g**). CXCL2 was present in very low concentrations in ^LPS^MSC-CM and ^LPS^NCSC-CM (**h**). CCL2 was more concentrated in NCSC-CM than in MSC-CM, but increased in both groups when prestimulated with LPS (**i**). TIMP-1 was equally secreted in all conditions (**j**), while M-CSF (**k**) and CXCL10 (**l**) were present in variable concentrations. (*n* = 5 to 7, one-way ANOVA and HSD post-test, *p < 0.05, **p < 0.01, ***p < 0.001). For values, see Additional file 1: Table S1. *MSC* mesenchymal stem cell, *NCSC* neural crest stem cell, *DMEM* Dulbecco’s modified Eagle’s medium, *LPS* lipopolysaccharide, *ELISA* enzyme-linked immunosorbent assay, *G-CSF* granulocyte colony-stimulating factor, *CXCL* C-X-C motif ligand, *CCL* C-C motif ligand, *IL-6* interleukin-6, *TIMP1*, tissue inhibitor of metalloproteinases 1, *ANOVA* analysis of variance, *HSD* honestly significant difference

Taking into account that MSCs and NCSCs seem to act quite early on their neighboring tissue and lead to an increased motor recovery as early as five days post-injury without integrating the host tissue, we hypothesized that cells could exert secretome-related activities. As previously mentioned, BMSCs can exert tissue repair by modulating diverse physiopathological events through the secretion of a wide variety of molecules, including cytokines and chemokines that are able to modulate immune cell recruitment and activity. In order to analyze the secretome of MSC_mix_ and NCSC_mix_, we collected serum-free culture medium (DMEM) in which equivalent numbers of cells were incubated for 24 h [MSC- and NCSC-conditioned media (CM)]. Two sets of conditions were compared: 1) MSC- or NCSC-CM without stimulation; and 2) ^LPS^MSC- or ^LPS^NCSC-CM from cells that were prestimulated with 1 μg/mL of lipopolysaccharide (LPS), for 24 h (Fig. 4a). Indeed, it is well known that LPS is able to induce the release of chemoattractant cytokines or chemokines from different cell types [45], and it was therefore interesting to mimic an inflammatory stimulus and analyze its effect on MSC and NCSC secretomes. Before testing LPS effects on MSCs and NCSCs, we confirmed from a previous microarray experiment that both cell types expressed genes coding for TLR4, and other downstream effectors (GSE30419), allowing these cells to properly respond to LPS stimulation [12].

We used a *Mouse Cytokine Array* to detect the different cytokines and chemokines secreted by MSCs and NCSCs. This qualitative analysis allowed us to observe that MSCs secreted a wide variety of cytokines such as CXCL10, CXCL1, M-CSF, CCL2, CXCL2, CCL5, CXCL12, and TIMP-1. LPS pre-stimulation boosted the secretion of some of these proteins and induced the secretion of CCL1 and IL-6 in ^LPS^MSC-CM. Conversely, we noticed that the secretome of NCSC_mix_ was not as enriched and that LPS stimulation did not induce such high modifications; still ,we observed the presence of CXCL10, CXCL1, M-CSF, CCL2 and TIMP-1 in NCSC-CM, and ^LPS^NCSC-CM (Fig. 4b).

ELISA arrays were then performed to obtain quantitative confirmation of those results (Fig. 2c-l). We observed that when stimulated with LPS, MSC_mix_ drastically increase the secretion of G-CSF, CXCL1, CCL5, CCL2, and IL-6. CXCL12 was exclusively secreted by MSC_mix_, but was not modified by LPS pre-treatment. When stimulated by LPS, both cell types secreted slight amounts of CXCL2. NCSC_mix_ secreted CCL2 (more than MSC_mix_ in basal conditions), which was further increased by LPS pre-conditioning. TIMP-1 was detected in high concentrations, both in MSC-CM and NCSC-CM, either with or without LPS pre-stimulation. Finally, M-CSF and CXCL10 were also detected, but in variable amounts (*n* = 5 to 7, one-way ANOVA and HSD post-test, *p < 0.05, **p < 0.01, ***p < 0.001). Values are presented in Additional file 1: Table S1.

### MSC and NCSC secretomes stimulate the metabolic activity and migration of RAW264.7 macrophages *in vitro*

**Fig. 5:**
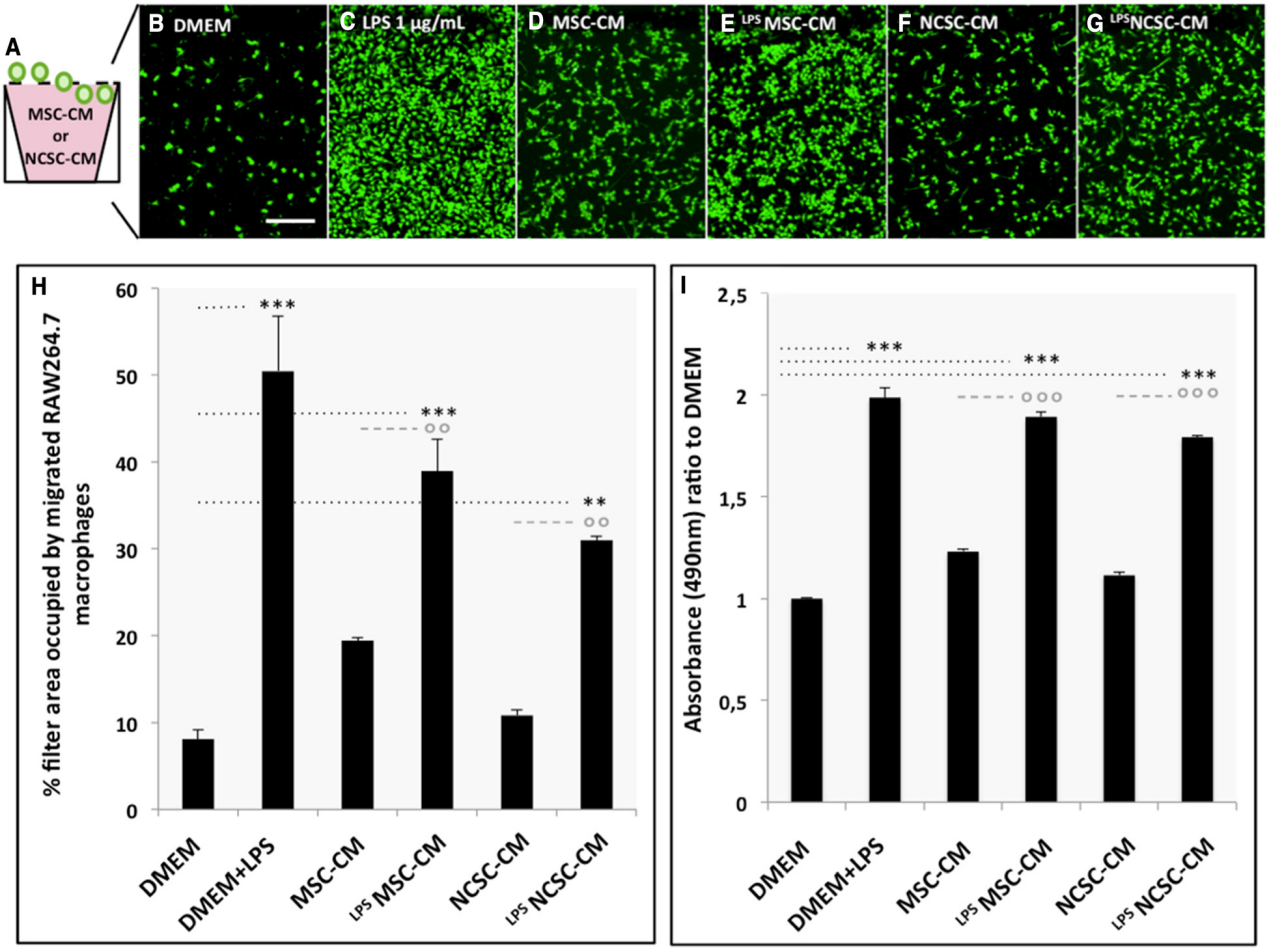
Effects of MSC- and NCSC-secretome on RAW264.7 macrophages migration and metabolic activity *in vitro*. We evaluated the chemoattractive properties of MSC and NCSC secretome by placing RAW264.7 macrophages on the top of a 5 μm-filter, with MSC-CM, ^LPS^MSC-CM, NCSC-CM or ^LPS^NCSC-CM in the bottom chamber (**a**). After 20 h, migration rate was assessed by the evaluation of the filter area occupied by macrophages. Passive migration was observed in response to serum-free DMEM (**b**, **h**), but drastically increased in response to LPS (**c**, **h**). MSC-CM slightly increased macrophage migration (**d**, **h**), but ^LPS^MSC-CM was more efficient (**e, h**). NCSC-CM did not significantly induce macrophage migration (**f, h**), while ^LPS^NCSC-CM also increased it (**g**, **h**). MTS metabolic assay also showed that ^LPS^MSC-CM and ^LPS^NCSC-CM increased the metabolic activity of RAW264.7 cells **i** (*n* = 3, one-way ANOVA and HSD post-test, **p < 0.01, ***p < 0.001). Scale bar = 50 μm. *MSC* mesenchymal stem cell, *NCSC* neural crest stem cell, *CM* conditioned medium, *LPS* lipopolysaccharide, *DMEM* Dulbecco’s modified Eagle’s medium, *ANOVA* analysis of variance, *HSD* honestly significant difference

In order to determine if MSC- and NCSC-secreted chemokines could exert functional chemoattraction, we tested the effect of MSC-CM, ^LPS^MSC-CM, NCSC-CM and ^LPS^NCSC-CM on the migration of RAW264.7 macrophages. These cells were subjected to chemotaxis through 5 μm-pores (Fig. 5a), and we calculated the percentage of filter area that was occupied by migrated cells. After 20 h, we observed that few macrophages passively migrated across the filter in the presence of DMEM (8.12 ± 1.06 %, Fig. 5b, h). LPS (1 μg/mL) was used as a positive control and considerably increased the migration of macrophages (50.42 ± 6.37 %, Fig. 5c, h). In the presence of MSC-CM, the number of macrophages recovered on the bottom side of the filter was slightly increased compared with DMEM (19.44 ± 0.31 %, *p* = 0.1) (Fig. 5d, h), but massively enhanced in the presence of ^LPS^MSC-CM (38.93 ± 3.66 %, ***p < 0.001) (Fig. 3e, h). No significant chemoattractant effect was observed for NCSC-CM compared to the control condition (10.83 ± 0.47 %, p > 0.5) (Fig. 5f, h); however, a significant increase in macrophage migration was observed in the presence of ^LPS^NCSC-CM (30.98 ± 0.47 %, **p < 0.01) (Fig. 5g, h) (*n* = 3, one-way ANOVA and HSD post-test, **p < 0.01, ***p < 0.001). Interestingly, these observations matched our previous results, showing that NCSC_mix_, but especially MSC_mix_ secreted massive concentrations of chemokines when prestimulated with LPS. We also confirmed that ^LPS^MSC-CM and ^LPS^NCSC-CM increased the metabolic activity of RAW264.7 macrophages after 20 h, as attested by MTS enzymatic assay (Fig. 5i) (*n* = 3, one-way ANOVA and HSD post-test, **p < 0.01, ***p < 0.001). Note that RAW264.7 macrophages do not exhibit any modification in membrane marker expression or secretion in response to MSC-CM or NCSC-CM (Additional file 3: Figure S2).

Additionally, we observed that incubation with MSC-CM or NCSC-CM did not modify the phenotype of RAW264.7 macrophages, which all express CD68, CD206, arginase-1, Iba1, CX3CR1, and CD11b in basal conditions and in the presence of CMs. Likewise, MSC-CM and NCSC-CM did not change the secretion profile of RAW264.7 macrophages (Additional file 2: Figure S1).

With the help of this *in vitro* migration assay, we observed that LPS-stimulated MSC- and NCSC-secreted chemokines efficiently stimulate the metabolic activity and the migration of macrophages. These results induced us to hypothesize that once inside a lesioned spinal cord, MSCs and NCSCs would be able to sense inflammatory stimuli and secrete chemokines that recruit immune cells towards the injury site.

### MSCs seem to recruit immune cells in blood and spinal cord of mice 24 h to seven days after spinal cord injury

**Fig. 6:**
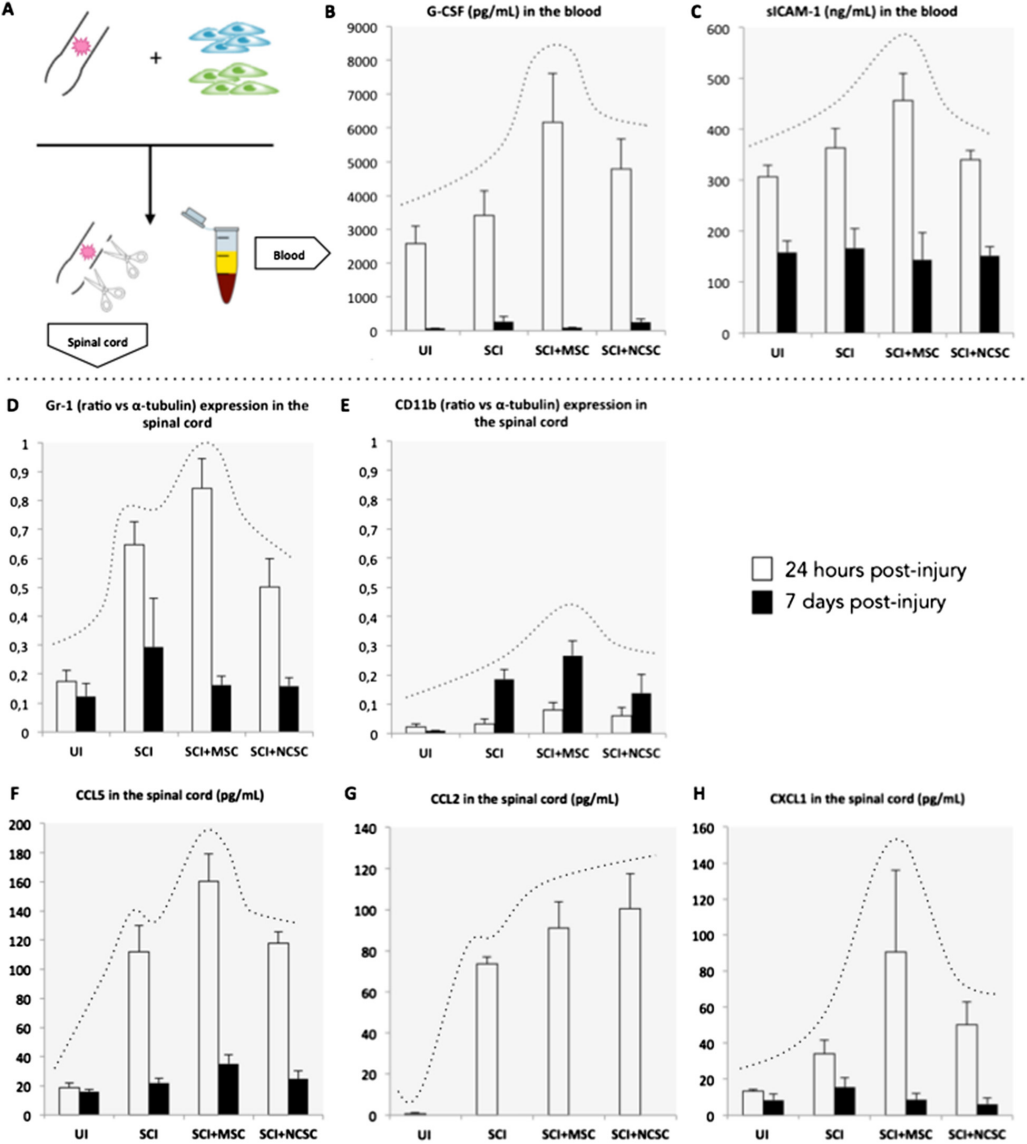
Early immune cell recruitment is slightly increased in the blood and spinal cord of MSC-grafted mice. We collected spinal cords and plasma of mice, 24 h (*white bars*) and seven days (*dark bars*) post-injury (**a**). We observed that the concentration of G-CSF in the plasma of MSC-grafted mice was slightly increased after 4 h compared to other groups (**b**), and the concentration of sICAM-1 as well (**c**), while both markers returned to low levels after seven days. Additionally, the expression of Gr-1 (reflecting the presence of granulocytes) was slightly increased in MSC-grafted spinal cords at 24 h, and returned to low levels after seven days (**d**) while the expression of CD11b increased at this delay (**e**). Interestingly, the concentrations of CCL5 and CXCL1 were also slightly boosted after 24 h in MSC-grafted spinal cords (**f**, **h**), while CCL2 tended to increase in both MSC- and NCSC-grafted groups (**g**). *MSC* mesenchymal stem cell, *G-CSF* granulocyte colony-stimulating factor, *sICAM1* soluble intercellular adhesion molecule, *CCL* C-C motif ligand, *CXCL* C-X-C motif ligand, *NCSC* neural crest stem cell

In order to determine the inflammatory events occurring acutely after the contusion that might explain our observations at 28 days post-injury, we collected the contused spinal cord tissue as well as the systemic blood of mice, 24 h and seven days following the intraspinal transplantation of MSC_mix_ and NCSC_mix_ (Fig. 6a). In order to investigate the molecular events that could reflect inflammatory reaction, we compared MSC_mix_- and NCSC_mix_-grafted SCI mice with PBS-injected SCI mice and uninjured (UI) mice (only subjected to laminectomy). The complexity of both blood and spinal cord extracts may explain the disparities that we observed between the different mice of each single group, giving rise to an unexpectedly high variability (*n* = 6 to 7, one-way ANOVA and HSD post-test, p > 0.05). Nevertheless, cytokine arrays (data not shown) and ELISA confirmations highlighted an increased plasmatic concentration of G-CSF (Fig. 6b) and sICAM-1 (Fig. 6c), 24 h post-injury, in mice receiving MSC_mix_. The same profile of data distribution was noticed after 24 h when looking at the level of Gr-1/Ly6G expression (mostly reflecting the presence of granulocytes) (Fig. 6d) as well as the concentration of CCL5 (Fig. 6f) and CXCL1 (Fig. 6h) in the spinal cord of MSC_mix_-grafted mice (*n* = 7, Spearman correlation test, *p < 0.05). Conversely, the concentration of CCL2 was slightly increased in both MSC_mix_- and NCSC_mix_-grafted mice (in accordance with the secretion of CCL2 by both cell types) (Fig. 6g). After seven days, the level of CD11b expression (mostly reflecting the presence of monocytes/macrophages) was also slightly higher in the MSC_mix_-injected group (Fig. 6e), whereas most of other parameters returned to initial levels.

## Discussion

For many years, bone marrow stromal stem cells (BMSCs) have been considered as useful tools for treating nervous system diseases in experimental research. Despite the demonstration of important benefits, critical information about the specific action of these cells in neurological lesions and repair is further required. As strongly suggested by the literature in the field, BMSCs act through paracrine secretion of growth factors, cytokines, chemokines, and other signaling molecules. This is of particular significance regarding physiopathological events that occur after a traumatic spinal cord injury (SCI), which encompass an intense inflammatory reaction that needs to be finely regulated.

In our study, we isolated two BMSC subpopulations, namely mesenchymal- (MSCs) and neural crest-derived stem cells (NCSCs), which we previously characterized in [12], and we observed that both MSCs and NCSCs significantly accelerate motor recovery when grafted intraspinally in mice with moderate SCI. This observation disproved our first hypothesis, namely that the variability of BMSC-based cell therapy results was due to the gathering of populations with diverse embryonic origins. We also observed that both MSC and NCSC graft induced a slight tissue sparing. Nevertheless, a precise identification and quantification of the axonal fibers that remain after lesion and/or are protected by grafted MSC and NCSC would more reliably correlate with improved motor recovery [46]. Furthermore, MSC and NCSC graft modified the lesion environment, as mainly depicted by the presence of arginase 1-expressing cells. Indeed, arginase-1 is commonly associated with an anti-inflammatory phenotype and metabolism [43] in immune cells, such as macrophages [47] or granulocytes [48]. We then formulated the hypothesis that MSC- and NCSC-induced motor recovery could be associated with a beneficial modulation of the inflammatory reaction. As we previously mentioned, peripheral blood monocytes are required for tissue repair after SCI [33], likely after being primed into an anti-inflammatory phenotype in the choroid plexus [34]. This hypothesis would then confirm recent data showing that BMSCs have beneficial effects in SCI mice by modulating the recruitment or the activity of alternatively activated macrophages [49, 50] and, therefore, enhancing tissue repair and motor behavior.

To support such a hypothesis, we showed that MSCs and NCSCs secrete various chemokines and cytokines, particularly when stimulated by *E. coli* lipopolysaccharide (LPS). We observed that after LPS stimulation, MSCs specifically secrete G-CSF, CXCL1, IL-6, CCL5, and CXCL12, which are almost absent in the NCSC secretome. These observations are in agreement with the high chemoattractant power of MSC secretome on RAW264.7 macrophages *in vitro*, since the role of these chemokines on immune cell recruitment is well described. Indeed, CXCL1, CXCL2, CCL5, and G-CSF are involved in the mobilization of neutrophils or monocytes in inflammatory conditions [51], including in brain tissue inflammation [52, 53], while CXCL12 enhances migration of diverse cell types in the organism [54]. IL-6 is a multifunctional neurotrophin and cytokine involved in neuronal survival, hematopoiesis, immune response, and systemic reaction to trauma [55]. Finally, CCL2 (also called monocyte chemoattractant protein 1 or MCP-1) is well known to stimulate macrophage migration under a wide variety of physiological or pathological conditions *in vitro* [56, 57] and *in vivo* [58, 59]. According to these data, we can infer that CXCL1, IL-6, G-CSF CXCL2, CXCL12, and CCL2 are together responsible for the chemoattractant action of MSC_mix_, which is further amplified when cells are prestimulated with LPS (correlated with increased concentrations of chemokines). On the other hand, *in vitro* assays have shown that only 10 ng/mL of CXCL1 is required to enhance migration of neutrophils [60], and we could assume that CXCL1 presence in ^LPS^MSC-CM could trigger chemotaxis by itself. Conversely, the NCSC secretome is less enriched, and the presence of CCL2 only may not be sufficient to induce chemoattraction at a similar level. Indeed, when used for macrophage migration assays, CCL2 is at least concentrated at 100 ng/mL [56, 57], and its concentration in NCSC-CM or ^LPS^NCSC-CM does not reach this threshold. Nevertheless, we obviously consider the possibility that the combination of CCL2 with low levels of CXCL1, CXCL2, and IL-6 would be able to induce migration of RAW264.7 cells.

Finally, we observed that stem cell transplantation (and more especially MSCs) seemed to enhance early inflammatory events in blood and spinal cord tissue, as reflected by high concentration of plasmatic G-CSF and sICAM-1 and the presence of CXCL1, CCL2, CCL5, and Gr-1-expressing granulocytes in the spinal cord 24 h post-SCI. Immune cell infiltration theoretically occurs in the first week following SCI [26, 61]; the signs of early amplified immune cell recruitment that we observed here were correlated with the early acceleration of motor recovery in MSC- and NCSC-grafted mice, which started around five days post-injury. It has been recently shown that neutrophil recruitment is mandatory in SCI repair [30, 62], so we could imagine that neutrophils could themselves have a specific activity in the lesioned spinal cord. Nonetheless, we also observed a slight increase in the number of macrophages in the lesion at 7 and 28 days post-injury, which could reflect macrophage recruitment that would be subsequent to neutrophil mobilization. Moreover, the presence of arginase-1-expressing cells, 28 days post-injury in the spinal cord and the slight tissue sparing seem to be remaining signs of a beneficial increased inflammatory reaction in the lesion area. It is now of huge importance to define the precise inflammatory sequence that is required for a suitable repair of the spinal cord tissue.

## Conclusion

In conclusion, our data suggest that pure populations of MSCs and NCSCs isolated from the adult bone marrow both have beneficial effects in SCI mice and the purification of these different subsets does not overtake the effects which are described with the whole BMSC population [5]. Altogether, this study confirms that great interest should be dedicated to the regulation of inflammatory reactions by exploring the pathways of chemokine-associated immune cell recruitment and activity [28] triggered by SCI and stem cell therapy. Despite the fact that the use of experimental models for SCI requires careful interpretation, new insights in the modulation of SCI-associated inflammation will further provide new tools and new therapeutic approaches that will finally improve recovery of SCI patients.

## Additional files

Additional file 1: Table S1. Negative controls of Iba1, GFAP and laminin immunostainings. (DOCX 54 kb) Additional file 2: Figure S1 Phenotypic profile of RAW264.7 macrophages is not modified in presence of MSC-CM and NCSC-CM. We observed that when placed in serum-free DMEM, or in MSC- or NCSC-CM, RAW264.7 all express arginase-1, CD11b, Iba1, CXC3CR1, CD68 or CD206. In addition, RAW264.7 cells do not secrete new cytokines in response to CM (new dots are associated with MSC-CM and NCSC-CM, see Fig. 4). (TIFF 1946 kb) Additional file 3: Figure S2. Phenotypic profile of RAW264.7 macrophages is not modified in presence of MSC-CM and NCSC-CM. We observed that when placed in serum-free DMEM, or in MSC- or NCSC-CM, RAW264.7 macrophages all express arginase 1, CD11b, Iba1, CX3CR1, CD206, CD68. Moreover, their secretion profile is not significantly modified. (TIFF 3075 kb)

## Abbreviations

BMS: Basso Mouse Scale
BMSC: Bone marrow stromal cell
CCL: C-C motif ligand
CD: Cluster of differentiation
CM: Conditioned medium
CNS: Central nervous system
CTG: Cell Tracker Green
CXCL: C-X-C motif ligand
DMEM: Dulbecco’s modified Eagle’s Medium
ELISA: Enzyme-linked immunosorbent assay
G-CSF: Granulocyte colony-stimulating factor
GFAP: Glial fibrillary acidic protein
Iba1: Ionized calcium-binding adapter molecule 1
IL: Interleukin
Ir: Immunoreactive
LPS: Lipopolysaccharide
M-CSF: Macrophage colony-stimulating factor
MSC: Mesenchymal stem cell
NCSC: Neural crest stem cell
PBS: Phosphate-buffered saline
sICAM1: Soluble intercellular adhesion molecule
TIMP1: Tissue inhibitor of metalloproteinases 1

## References

[1] Neirinckx V, Coste C, Rogister B, Wislet-Gendebien S (2013). Concise review: adult mesenchymal stem cells, adult neural crest stem cells, and therapy of neurological pathologies: a state of play. Stem Cells Transl Med.

[2] Singer NG, Caplan AI (2011). Mesenchymal stem cells: mechanisms of inflammation. Annu Rev Pathol.

[3] Crigler L, Robey RC, Asawachaicharn A, Gaupp D, Phinney DG (2006). Human mesenchymal stem cell subpopulations express a variety of neuro-regulatory molecules and promote neuronal cell survival and neuritogenesis. Exp Neurol.

[4] Hsieh JY, Wang HW, Chang SJ, Liao KH, Lee IH, Lin WS (2013). Mesenchymal stem cells from human umbilical cord express preferentially secreted factors related to neuroprotection, neurogenesis, and angiogenesis. PLoS One.

[5] Neirinckx V, Cantinieaux D, Coste C, Rogister B, Franzen R, Wislet-Gendebien S (2014). Concise review: spinal cord injuries: how could adult mesenchymal and neural crest stem cells take up the challenge?. Stem Cells.

[6] Karamouzian S, Nematollahi-Mahani SN, Nakhaee N, Eskandary H (2012). Clinical safety and primary efficacy of bone marrow mesenchymal cell transplantation in subacute spinal cord injured patients. Clin Neurol Neurosurg.

[7] Jiang Y, Jahagirdar BN, Reinhardt RL, Schwartz RE, Keene CD, Ortiz-Gonzalez XR (2002). Pluripotency of mesenchymal stem cells derived from adult marrow. Nature.

[8] D’Ippolito G, Diabira S, Howard GA, Menei P, Roos BA, Schiller PC (2004). Marrow-isolated adult multilineage inducible (MIAMI) cells, a unique population of postnatal young and old human cells with extensive expansion and differentiation potential. J Cell Sci.

[9] Dennis JE, Charbord P (2002). Origin and differentiation of human and murine stroma. Stem Cells.

[10] Morikawa S, Mabuchi Y, Niibe K, Suzuki S, Nagoshi N, Sunabori T (2009). Development of mesenchymal stem cells partially originate from the neural crest. Biochem Biophys Res Commun.

[11] Nagoshi N, Shibata S, Kubota Y, Nakamura M, Nagai Y, Satoh E (2008). Ontogeny and multipotency of neural crest-derived stem cells in mouse bone marrow, dorsal root ganglia, and whisker pad. Cell Stem Cell.

[12] Wislet-Gendebien S, Laudet E, Neirinckx V, Alix P, Leprince P, Glejzer A (2012). Mesenchymal stem cells and neural crest stem cells from adult bone marrow: characterization of their surprising similarities and differences. Cell Mol Life Sci.

[13] Neirinckx V, Marquet A, Coste C, Rogister B, Wislet-Gendebien S (2013). Adult bone marrow neural crest stem cells and mesenchymal stem cells are not able to replace lost neurons in acute MPTP-lesioned mice. PLoS One.

[14] Khoo ML, Tao H, Meedeniya AC, Mackay-Sim A, Ma DD (2011). Transplantation of neuronal-primed human bone marrow mesenchymal stem cells in hemiparkinsonian rodents. PLoS One.

[15] Blandini F, Cova L, Armentero MT, Zennaro E, Levandis G, Bossolasco P (2010). Transplantation of undifferentiated human mesenchymal stem cells protects against 6-hydroxydopamine neurotoxicity in the rat. Cell Transplant.

[16] Chao YX, He BP, Tay SS (2009). Mesenchymal stem cell transplantation attenuates blood brain barrier damage and neuroinflammation and protects dopaminergic neurons against MPTP toxicity in the substantia nigra in a model of Parkinson’s disease. J Neuroimmunol.

[17] Kamada T, Koda M, Dezawa M, Anahara R, Toyama Y, Yoshinaga K (2011). Transplantation of human bone marrow stromal cell-derived Schwann cells reduces cystic cavity and promotes functional recovery after contusion injury of adult rat spinal cord. Neuropathology.

[18] Sasaki M, Radtke C, Tan AM, Zhao P, Hamada H, Houkin K (2009). BDNF-hypersecreting human mesenchymal stem cells promote functional recovery, axonal sprouting, and protection of corticospinal neurons after spinal cord injury. J Neurosci.

[19] Teixeira FG, Carvalho MM, Sousa N, Salgado AJ (2013). Mesenchymal stem cells secretome: a new paradigm for central nervous system regeneration?. Cell Mol Life Sci.

[20] Paul G, Anisimov SV (2013). The secretome of mesenchymal stem cells: potential implications for neuroregeneration. Biochimie.

[21] Cantinieaux D, Quertainmont R, Blacher S, Rossi L, Wanet T, Noel A (2013). Conditioned medium from bone marrow-derived mesenchymal stem cells improves recovery after spinal cord injury in rats: an original strategy to avoid cell transplantation. PLoS One.

[22] Akyurekli C, Le Y, Richardson RB, Fergusson D, Tay J, Allan DS (2015). A systematic review of preclinical studies on the therapeutic potential of mesenchymal stromal cell-derived microvesicles. Stem Cell Rev.

[23] Bernardo ME, Fibbe WE (2013). Mesenchymal stromal cells: sensors and switchers of inflammation. Cell Stem Cell.

[24] Prockop DJ, Oh JY (2012). Mesenchymal stem/stromal cells (MSCs): role as guardians of inflammation. Mol Ther.

[25] Le Blanc K, Rasmusson I, Sundberg B, Gotherstrom C, Hassan M, Uzunel M (2004). Treatment of severe acute graft-versus-host disease with third party haploidentical mesenchymal stem cells. Lancet.

[26] Beck KD, Nguyen HX, Galvan MD, Salazar DL, Woodruff TM, Anderson AJ (2010). Quantitative analysis of cellular inflammation after traumatic spinal cord injury: evidence for a multiphasic inflammatory response in the acute to chronic environment. Brain.

[27] Bracken MB (1990). Methylprednisolone in the management of acute spinal cord injuries. Med J Aust.

[28] Donnelly DJ, Popovich PG (2008). Inflammation and its role in neuroprotection, axonal regeneration and functional recovery after spinal cord injury. Exp Neurol.

[29] Yong VW, Rivest S (2009). Taking advantage of the systemic immune system to cure brain diseases. Neuron.

[30] Neirinckx V, Coste C, Franzen R, Gothot A, Rogister B, Wislet S (2014). Neutrophil contribution to spinal cord injury and repair. J Neuroinflammation.

[31] David S, Kroner A (2011). Repertoire of microglial and macrophage responses after spinal cord injury. Nat Rev Neurosci.

[32] Knoller N, Auerbach G, Fulga V, Zelig G, Attias J, Bakimer R (2005). Clinical experience using incubated autologous macrophages as a treatment for complete spinal cord injury: phase I study results. J Neurosurg Spine.

[33] Shechter R, London A, Varol C, Raposo C, Cusimano M, Yovel G (2009). Infiltrating blood-derived macrophages are vital cells playing an anti-inflammatory role in recovery from spinal cord injury in mice. PLoS Med.

[34] Shechter R, Miller O, Yovel G, Rosenzweig N, London A, Ruckh J (2013). Recruitment of beneficial M2 macrophages to injured spinal cord is orchestrated by remote brain choroid plexus. Immunity.

[35] Kigerl KA, Gensel JC, Ankeny DP, Alexander JK, Donnelly DJ, Popovich PG (2009). Identification of two distinct macrophage subsets with divergent effects causing either neurotoxicity or regeneration in the injured mouse spinal cord. J Neurosci.

[36] Jiang X, Rowitch DH, Soriano P, McMahon AP, Sucov HM (2000). Fate of the mammalian cardiac neural crest. Development.

[37] Danielian PS, Muccino D, Rowitch DH, Michael SK, McMahon AP (1998). Modification of gene activity in mouse embryos in utero by a tamoxifen-inducible form of Cre recombinase. Curr Biol.

[38] Soriano P (1999). Generalized lacZ expression with the ROSA26 Cre reporter strain. Nat Genet.

[39] Neirinckx V, Rogister B, Franzen R, Wislet-Gendebien S (2014). Bone marrow stromal stem cells transplantation in mice with acute spinal cord injury. Methods Mol Biol.

[40] Lockard I, Reers BL (1962). Staining tissue of the central nervous system with luxol fast blue and neutral red. Stain Technol.

[41] Basso DM, Fisher LC, Anderson AJ, Jakeman LB, McTigue DM, Popovich PG (2006). Basso Mouse Scale for locomotion detects differences in recovery after spinal cord injury in five common mouse strains. J Neurotrauma.

[42] Ritfeld GJ, Oudega M. Bone marrow-derived mesenchymal stem cell transplant survival in the injured rodent spinal cord. J Bone Marrow Res. 2014;2(2).

[43] Popovic PJ, Zeh HJ, Ochoa JB (2007). Arginine and immunity. J Nutr.

[44] Ahn M, Lee C, Jung K, Kim H, Moon C, Sim KB (2012). Immunohistochemical study of arginase-1 in the spinal cords of rats with clip compression injury. Brain Res.

[45] Kopydlowski KM, Salkowski CA, Cody MJ, van Rooijen N, Major J, Hamilton TA (1999). Regulation of macrophage chemokine expression by lipopolysaccharide in vitro and in vivo. J Immunol.

[46] You SW, Chen BY, Liu HL, Lang B, Xia JL, Jiao XY (2003). Spontaneous recovery of locomotion induced by remaining fibers after spinal cord transection in adult rats. Restor Neurol Neurosci.

[47] Sica A, Mantovani A (2012). Macrophage plasticity and polarization: in vivo veritas. J Clin Invest.

[48] Fridlender ZG, Sun J, Kim S, Kapoor V, Cheng G, Ling L (2009). Polarization of tumor-associated neutrophil phenotype by TGF-beta: “N1” versus “N2” TAN. Cancer Cell.

[49] Nakajima H, Uchida K, Guerrero AR, Watanabe S, Sugita D, Takeura N (2012). Transplantation of mesenchymal stem cells promotes an alternative pathway of macrophage activation and functional recovery after spinal cord injury. J Neurotrauma.

[50] Busch SA, Hamilton JA, Horn KP, Cuascut FX, Cutrone R, Lehman N (2011). Multipotent adult progenitor cells prevent macrophage-mediated axonal dieback and promote regrowth after spinal cord injury. J Neurosci.

[51] Wengner AM, Pitchford SC, Furze RC, Rankin SM (2008). The coordinated action of G-CSF and ELR + CXC chemokines in neutrophil mobilization during acute inflammation. Blood.

[52] Johnson EA, Dao TL, Guignet MA, Geddes CE, Koemeter-Cox AI, Kan RK (2011). Increased expression of the chemokines CXCL1 and MIP-1alpha by resident brain cells precedes neutrophil infiltration in the brain following prolonged soman-induced status epilepticus in rats. J Neuroinflammation.

[53] Babcock AA, Kuziel WA, Rivest S, Owens T (2003). Chemokine expression by glial cells directs leukocytes to sites of axonal injury in the CNS. J Neurosci.

[54] Karin N (2010). The multiple faces of CXCL12 (SDF-1alpha) in the regulation of immunity during health and disease. J Leukoc Biol.

[55] Galiano M, Liu ZQ, Kalla R, Bohatschek M, Koppius A, Gschwendtner A (2001). Interleukin-6 (IL6) and cellular response to facial nerve injury: effects on lymphocyte recruitment, early microglial activation and axonal outgrowth in IL6-deficient mice. Eur J Neurosci.

[56] van Gils JM, Derby MC, Fernandes LR, Ramkhelawon B, Ray TD, Rayner KJ (2012). The neuroimmune guidance cue netrin-1 promotes atherosclerosis by inhibiting the emigration of macrophages from plaques. Nat Immunol.

[57] Lu X, Kang Y (2009). Chemokine (C-C motif) ligand 2 engages CCR2+ stromal cells of monocytic origin to promote breast cancer metastasis to lung and bone. J Biol Chem.

[58] Fuentes ME, Durham SK, Swerdel MR, Lewin AC, Barton DS, Megill JR (1995). Controlled recruitment of monocytes and macrophages to specific organs through transgenic expression of monocyte chemoattractant protein-1. J Immunol.

[59] Siebert H, Sachse A, Kuziel WA, Maeda N, Bruck W (2000). The chemokine receptor CCR2 is involved in macrophage recruitment to the injured peripheral nervous system. J Neuroimmunol.

[60] De Filippo K, Dudeck A, Hasenberg M, Nye E, van Rooijen N, Hartmann K (2013). Mast cell and macrophage chemokines CXCL1/CXCL2 control the early stage of neutrophil recruitment during tissue inflammation. Blood.

[61] Fleming JC, Norenberg MD, Ramsay DA, Dekaban GA, Marcillo AE, Saenz AD (2006). The cellular inflammatory response in human spinal cords after injury. Brain.

[62] Stirling DP, Liu S, Kubes P, Yong VW (2009). Depletion of Ly6G/Gr-1 leukocytes after spinal cord injury in mice alters wound healing and worsens neurological outcome. J Neurosci.

