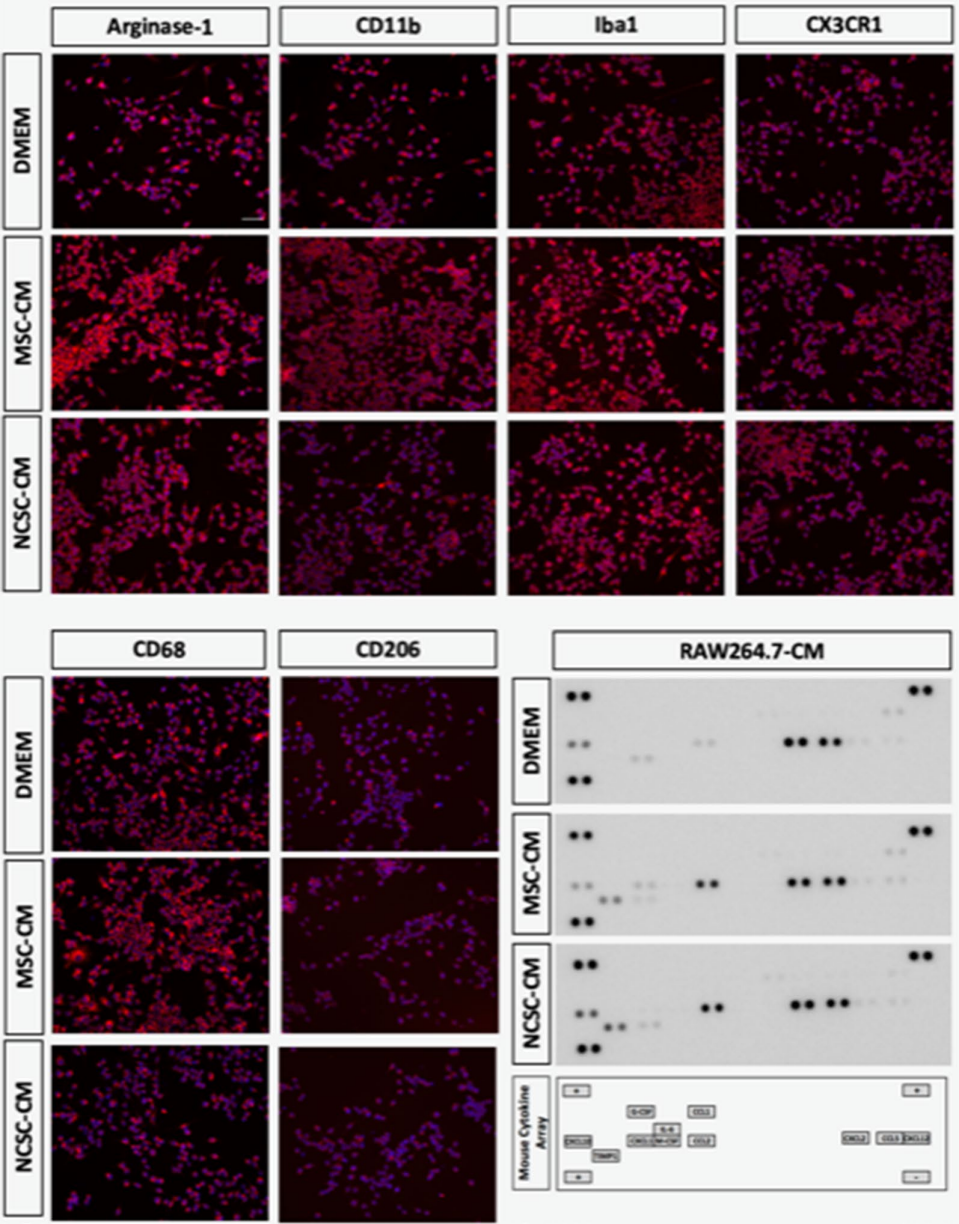# Correction to: Adult bone marrow mesenchymal and neural crest stem cells are chemoattractive and accelerate motor recovery in a mouse model of spinal cord injury

**DOI:** 10.1186/s13287-021-02534-z

**Published:** 2021-09-22

**Authors:** Virginie Neirinckx, Gulistan Agirman, Cécile Coste, Alice Marquet, Valérie Dion, Bernard Rogister, Rachelle Franzen, Sabine Wislet

**Affiliations:** 1 Groupe Interdisciplinaire de Génoprotéomique Appliquée (GIGA), Neurosciences Research Center, Unit of Nervous system disorders and treatment, University of Liège, Tour de Pathologie 2, Avenue de l’Hôpital, 1, 4000 Liège, Belgium; 2 GIGA, Development, Stem Cells and Regenerative Medicine Research Center, University of Liège, Liège, Belgium; 3 Neurology Department, University Hospital, Liège, Belgium

## Correction to: Stem Cell Research & Therapy (2015) 6:211 10.1186/s13287-015-0202-2

Following publication of the original article [1], it has been raised to authors’ attention that the manuscript included misused or duplicated elements.

**Figure 1A**: PCR illustration for PGK-Neomycin and Actin was reused from previous publication [2] without proper citation.

**Figure S1:** Iba1_DMEM and Iba1_NCSC-CM as well as CD68_ and CD206_NCSC-CM conditions were duplicated.

Corrected Figure 1A and S1 are illustrated ahead. Corrections were performed based on the experimental confirmation of the original data. Of note, neither results nor conclusions drawn from this study are modified.

In addition, careful proofreading of the manuscript highlighted that titles of Figure S2 and Table S3 were erroneous, and were then corrected as follows:

- Figure S2: Negative controls of Iba1, GFAP and laminin immunostainings.
- Table S1: ELISA assays values.

The authors sincerely apologize for any inconvenience this may have caused to Stem Cell Research & Therapy, as well as to the scientific community.

**Figure 1** 
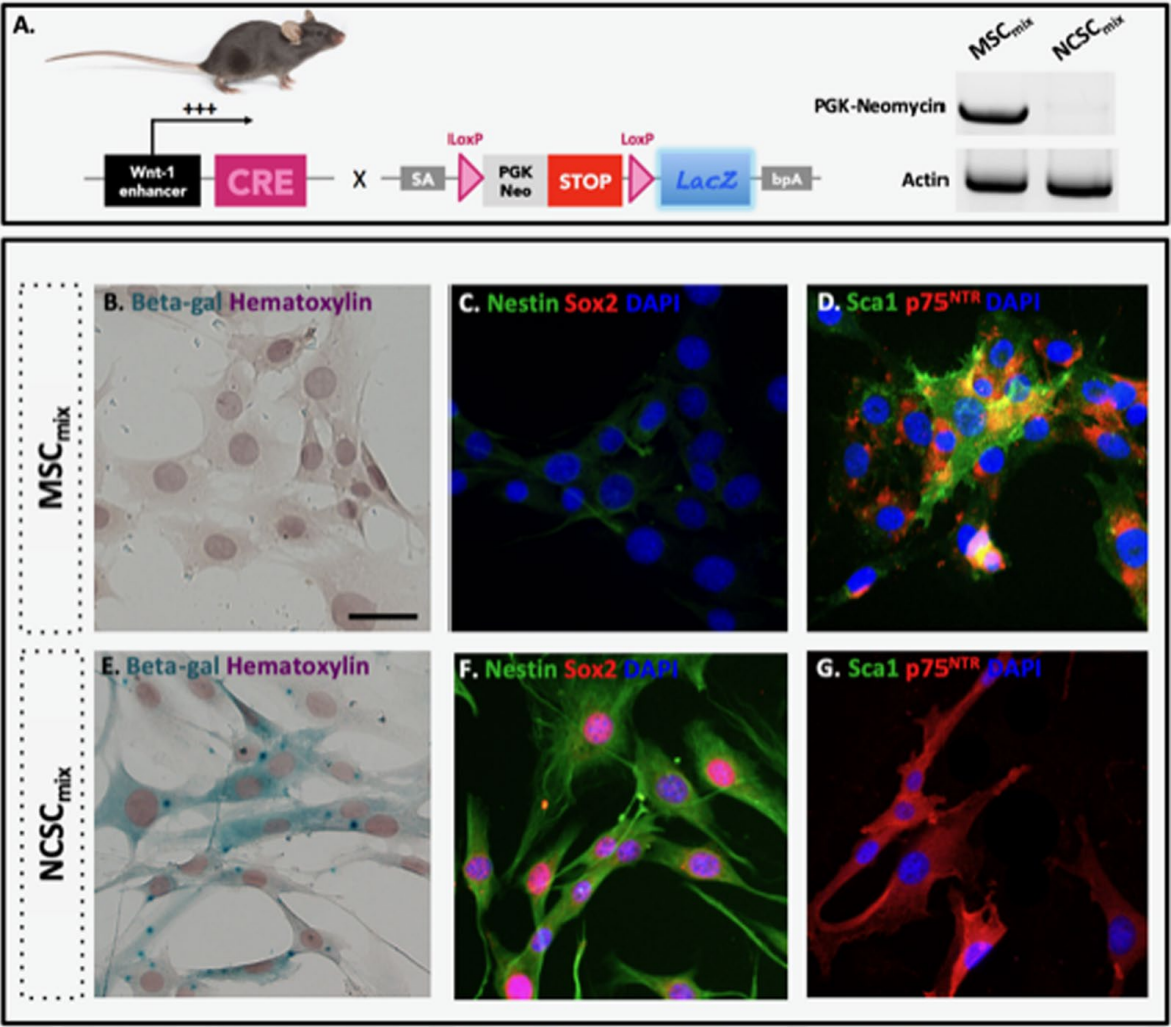


**Figure S1**: