# Supplementary material for: Adult bone marrow mesenchymal and neural crest stem cells are chemoattractive and accelerate motor recovery in a mouse model of spinal cord injury
Source: Stem Cell Res Ther. 2015 Nov 4;6:211. doi: 10.1186/s13287-015-0202-2 (PMC4632651; doi:10.1186/s13287-015-0202-2)
Supplement: Additional file 1: Table S1. — Negative controls of Iba1, GFAP and laminin immunostainings. (DOCX 54 kb) [file 13287_2015_202_MOESM1_ESM.docx]

| *(n=5 to 7)*  *pg/mL* | **MSC** | **MSC LPS** | **NCSC** | **NCSC LPS** | **DMEM** | **DMEM LPS** |
| --- | --- | --- | --- | --- | --- | --- |
| ***G-CSF*** | 1,27 ± 0,28 | 72,62 ± 26,87 | 0,22 ± 0,22 | 0,44 ± 0,28 | 0,00 ± 0,00 | 0,00 ± 0,00 |
| ***CXCL1*** | 421,03 ± 97,38 | 12320,53 ± 1261,05 | 68,76 ± 8,33 | 1190,81 ± 241,8 |  |  |
| ***CCL2*** | 3189,75 ± 598,10 | 14410,88 ± 369,95 | 7797,84 ± 1223,84 | 12523,70 ± 1344,35 |  |  |
| ***CCL5*** | 111,69 ± 29,54 | 2443,04 ± 591,64 | 10,09 ± 2,39 | 33,80 ± 6,26 |  |  |
| ***TIMP-1*** | 8455,75 ± 818,87 | 10193,25 ± 631,35 | 8916,67 ± 918,19 | 9152,82 ± 583,44 |  |  |
| ***CXCL12*** | 1650,00 ± 490,00 | 1680,00 ± 670,00 | 30 ± 1,00 | 0,00 ± 0,00 |  |  |
| ***IL-6*** | 4,58 ± 0,70 | 108,47 ± 15,81 | 3,49 ± 0,56 | 16,46 ± 3,68 |  |  |
| ***M-CSF*** | 123,04 ± 29,68 | 283,96 ± 92,64 | 26,89 ± 9,27 | 110,92 ± 33,52 |  |  |
| ***CXCL2*** | 0,10 ± 0,08 | 1,69 ± 0,40 | 0,04 ± 0,04 | 3,84 ± 1,23 |  |  |
| ***CXCL10*** | 24,65 ± 20,84 | 27,67 ± 10,46 | 81,18 ± 33,28 | 151,24 ± 50,32 |  |  |
