# Supplementary figures and images for: Adult bone marrow mesenchymal and neural crest stem cells are chemoattractive and accelerate motor recovery in a mouse model of spinal cord injury
Source: Stem Cell Res Ther. 2015 Nov 4;6:211. doi: 10.1186/s13287-015-0202-2 (PMC4632651; doi:10.1186/s13287-015-0202-2)

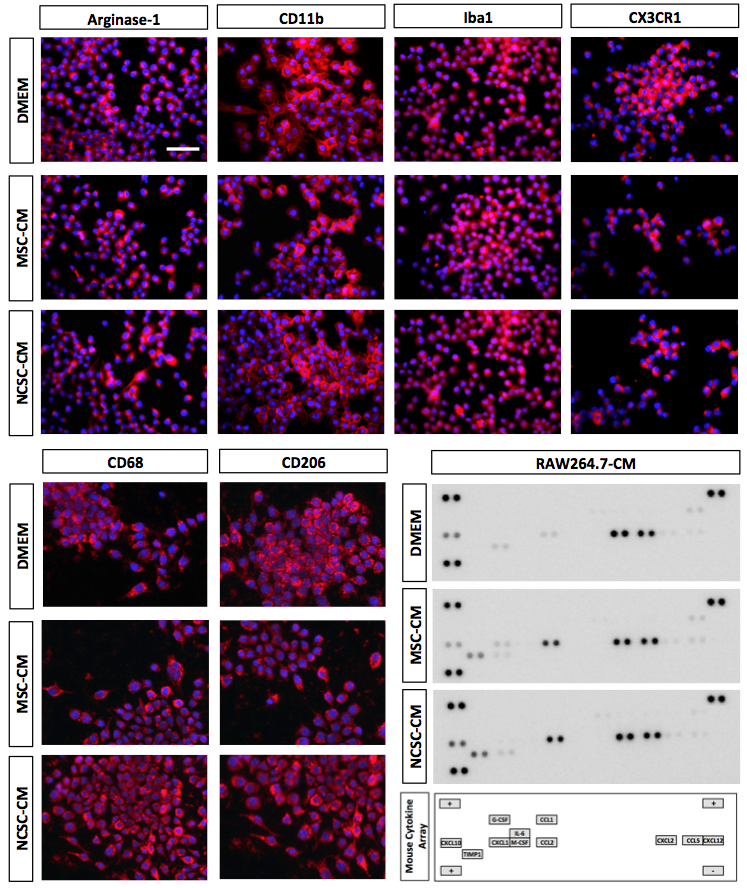

Supplement: Additional file 2: Figure S1 — Phenotypic profile of RAW264.7 macrophages is not modified in presence of MSC-CM and NCSC-CM. We observed that when placed in serum-free DMEM, or in MSC- or NCSC-CM, RAW264.7 all express arginase-1, CD11b, Iba1, CXC3CR1, CD68 or CD206. In addition, RAW264.7 cells do not secrete new cytokines in response to CM (new dots are associated with MSC-CM and NCSC-CM, see Fig. 4). (TIFF 1946 kb) [file 13287_2015_202_MOESM2_ESM.tiff]

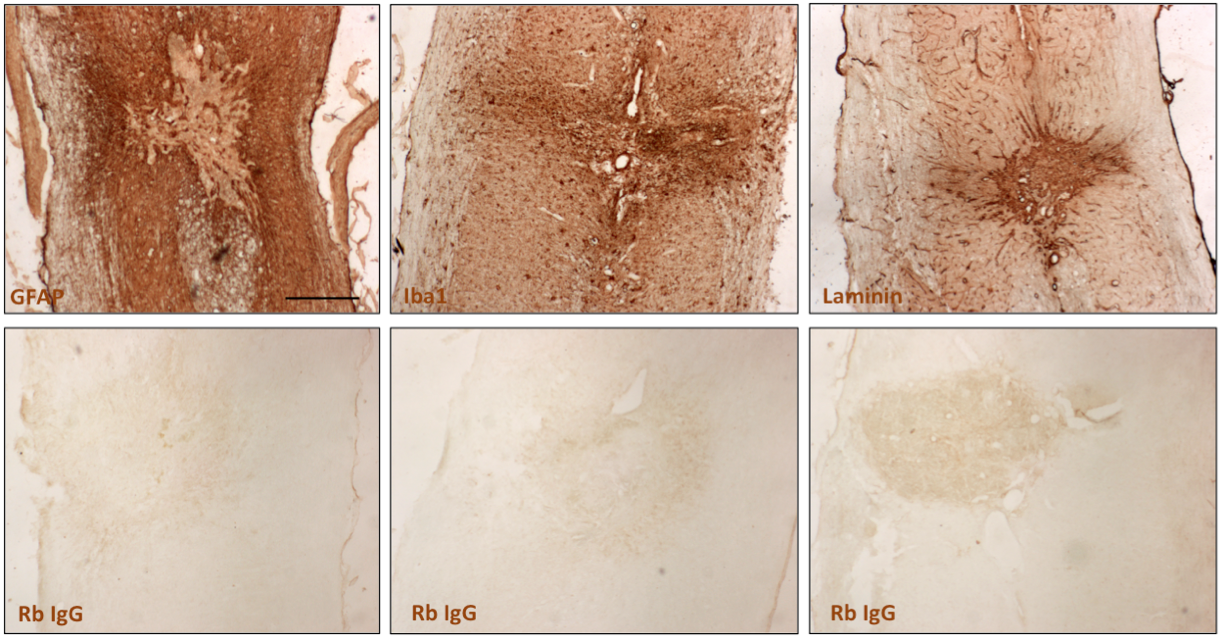

Supplement: Additional file 3: Figure S2. — Phenotypic profile of RAW264.7 macrophages is not modified in presence of MSC-CM and NCSC-CM. We observed that when placed in serum-free DMEM, or in MSC- or NCSC-CM, RAW264.7 macrophages all express arginase 1, CD11b, Iba1, CX3CR1, CD206, CD68. Moreover, their secretion profile is not significantly modified. (TIFF 3075 kb) [file 13287_2015_202_MOESM3_ESM.tiff]
